# The Role of Myeloid Cell Activation and Arginine Metabolism in the Pathogenesis of Virus-Induced Diseases

**DOI:** 10.3389/fimmu.2014.00428

**Published:** 2014-09-08

**Authors:** Kristina S. Burrack, Thomas E. Morrison

**Affiliations:** ^1^Department of Immunology and Microbiology, University of Colorado School of Medicine, Aurora, CO, USA

**Keywords:** arginase, iNOS, viral pathogenicity, macrophages, immunity, cellular

## Abstract

When an antiviral immune response is generated, a balance must be reached between two opposing pathways: the production of proinflammatory and cytotoxic effectors that drive a robust antiviral immune response to control the infection and regulators that function to limit or blunt an excessive immune response to minimize immune-mediated pathology and repair tissue damage. Myeloid cells, including monocytes and macrophages, play an important role in this balance, particularly through the activities of the arginine-hydrolyzing enzymes nitric oxide synthase 2 (Nos2; iNOS) and arginase 1 (Arg1). Nitric oxide (NO) production by iNOS is an important proinflammatory mediator, whereas Arg1-expressing macrophages contribute to the resolution of inflammation and wound repair. In the context of viral infections, expression of these enzymes can result in a variety of outcomes for the host. NO has direct antiviral properties against some viruses, whereas during other virus infections NO can mediate immunopathology and/or inhibit the antiviral immune response to promote chronic infection. Arg1 activity not only has important wound healing functions but can also inhibit the antiviral immune response during some viral infections. Thus, depending on the specific virus and the tissue(s) involved, the activity of both of these arginine-hydrolyzing enzymes can either exacerbate or limit the severity of virus-induced disease. In this review, we will discuss a variety of viral infections, including HIV, SARS-CoV, LCMV, HCV, RSV, and others, where myeloid cells influence the control and clearance of the virus from the host, as well as the severity and resolution of tissue damage, via the activities of iNOS and/or Arg1. Clearly, monocyte/macrophage activation and arginine metabolism will continue to be important areas of investigation in the context of viral infections.

## Introduction

Tissue-resident and monocyte-derived macrophages are innate immune cells that play a key role in normal tissue homeostasis, presentation of foreign and self antigens following infection or injury, pathogen clearance, and resolution of inflammation and wound healing. Depending on the microenvironment, macrophages can be programed to adopt a variety of proinflammatory, regulatory, resolving, and immunosuppressive activation phenotypes, particularly *in vivo*. These activation states exist as a complex continuum of overlapping phenotypes; however, macrophage subsets with distinct functions have been defined (1). Macrophages are considered M1-polarized when stimulated by IFN-γ or Toll-like receptor (TLR) ligands, such as lipopolysaccharide (LPS), to express inducible nitric oxide synthase (iNOS; Nos2) and produce nitric oxide (NO). NOS enzymes metabolize l-arginine to citrulline and NO. NO is a short-lived gaseous messenger with physiological and pathological effects. Nanomolar concentrations of NO, generated by endothelial NOS and neuronal NOS, are important for maintaining homeostasis, regulating vasodilation, and for the aggregation, recruitment, and adhesion of platelets to the vascular endothelium. iNOS generates micromolar levels of NO that modulates various pathophysiological processes and is important for killing intracellular pathogens (2).

In contrast, M2-polarized macrophages result following stimulation of cells with a variety of stimuli, including type 2 cytokines such as interleukin (IL)-4 or IL-13. M2-polarized macrophages express a distinct l-arginine-metabolizing enzyme, arginase 1 (Arg1), which hydrolyzes l-arginine to l-ornithine and urea. l-Ornithine can be further metabolized to polyamines, which participate in a variety of fundamental cellular functions (e.g., proliferation, cell membrane transport), and l-proline, which is an essential component of collagen. In addition to playing important roles in defense against extracellular parasites and tissue repair, Arg1 expression and activity in myeloid cells have emerged as a major regulator of innate and adaptive immune responses (3). Other M2-like suppressive or anti-inflammatory macrophages include myeloid-derived suppressor cells (MDSCs) and tumor-associated macrophages (TAMs). MDSCs are considered to be an immature population of myeloid cells, including both monocyte-like (GR-1/Ly-6C^+^) and neutrophil-like (GR-1/Ly-6G^+^) populations, associated with tumors or infections that suppress proinflammatory responses (4, 5). Depending on the context, MDSCs have been shown to mediate their suppressive activity via NO- and/or Arg1-dependent mechanisms. Importantly, macrophages are not permanently programed, but are considered “plastic” – that is, macrophages have been shown to change activation phenotypes depending on the local environment.

Although the M1/iNOS and M2/Arg1 division is generally appropriate, Arg1 can be induced in M1-like macrophages under certain conditions. Thus, due to the spectrum of activation states for macrophages, a framework for macrophage-activation nomenclature was recently suggested (6). In an attempt to avoid confusion in this review, we focused on the specific effects of the l-arginine metabolizing enzymes iNOS or Arg1 on the pathogenesis of viral infections, noting other activation markers where appropriate.

Increasing evidence suggests that myeloid cell programing, iNOS, and Arg1 contribute to the pathogenesis of numerous virus infections, suggesting that therapies that target these cells and pathways may be beneficial for the treatment of some virus diseases. In this review, we highlight recent studies of viral infections where myeloid cell polarization – resulting in expression of iNOS or Arg1 – contribute to viral control or the development of chronic virus infection and mediate the resolution of tissue damage or cause immunopathology.

## NO Production Can be Beneficial during Virus Infection

NO has antimicrobial activity against a number of bacteria, parasites, and fungi (7, 8). Additionally, NO has been shown to have direct antiviral effects *in vitro* and/or *in vivo* against several viruses, including DNA viruses such as herpes simplex virus type-1 (HSV-1), ectromelia virus (EV), and vaccinia virus (VV) (9, 10), as well as some RNA viruses such as vesicular stomatitis virus (VSV) (11), Japanese encephalitis virus (JEV) (12), dengue virus (DENV) (13), and coxsackievirus (Table 1) (14–17). There are several advantages of using NO as an antiviral agent. For instance, unlike complement and antibody, NO can readily pass through cellular membranes into neighboring cells as well as some viruses. Additionally, NO is likely to act on a variety of both viral and virally exploited cellular targets, inhibiting viral replication as well as limiting the capacity of viruses to develop resistance. Lastly, the effect of NO is independent of immune recognition of the infected cell, in contrast to that of antiviral lymphocytes, which could be important in virus-infected cells where expression of MHC class I molecules may be downregulated and in some virally infected tissues such as the brain where expression of MHC class I and II molecules is limited.

**Table 1:** **Differential roles for the l-arginine-hydrolyzing enzymes iNOS and Argl in virus-induced diseases**.

		iNOS/NO	Reference		Arg1	Reference
**Beneficial for host**	**Antiviral**	Ectromelia virus (EV)	(9, 10, 18)	**Tissue repair/regeneration**	Respiratory syncytial virus (RSV)	(19–21)
		Herpes simplex virus-1 (HSV-1)	(9, 10, 22–26)		Influenza	(27)
		Cytomegalovirus (CMV)^a^	(28, 29)		Coxsackievirus B3 (CVB3)^d^	(30, 31)
		Vaccinia virus (VV)	(9, 10, 32)			
		Marek’s disease virus (MDV)^b^	(33 –35)			
		Hepatitis B virus (HBV)	(36)			
		Vesicular stomatitis virus (VSV)	(11)			
		Japanese encephalitis virus (JEV)	(12)			
		Dengue virus (DENV)	(13, 37–41)			
		West Nile virus (WNV)	(42)			
		Sindbis virus (SINV)	(43)			
		Reovirus (T3A strain)	(44)			
		Coxsackievirus B3 and B4 (CVB3, CVB4)	(14–17, 91)			
**Detrimental to host**	**Immunopathologic**	Influenza	(45–55)	**Immunopathologic**	SARS-CoV	(56, 57)
		HSV-1	(58–60)		Murine γ-herpesvirus-68 (MHV-68)^e^	(61 –63)
		Feline immunodeficiency virus	(64, 65)	
	**Promotes viral persistence**	Cytomegalovirus (CMV)^c^	(66)	**Promotes viral persistence**	LCMV clone 13	(67)
		rJ2.2 strain of mouse hepatitis virus (neurotropic coronavirus)	(68)		Ross River virus (RRV), chikungunya virus (CHIKV)	(69)
					Marek’s disease virus (MDV)^f^	(35)
		Lymphocytic choriomeningitis virus (LCMV) clone 13	(70)		Hepatitis C virus (HCV)	(71)
					Human immunodeficiency virus (HIV)	(72–77)
					Hepatitis B virus (HBV)	(78–80)
					Influenza	(81, 82)
					Human papillomavirus (HPV)^g^	(83)

*^a^HCMV, *in vitro*; MCMV, in 129/Sv/Ev x C57BL/6 F1 mice*.

*^b^For resistant chickens*.

*^c^MCMV, in BALB/c mice*.

*^d^In female BALB/c mice*.

*^e^In *Ifngr*^  −/−^ mice*.

*^f^For susceptible chickens*.

*^g^Mouse model of HPV-induced cancer*.

In initial studies *in vitro*, inhibition of EV, VV, and HSV-1 replication in mouse RAW 264.7 macrophages and in primary mouse macrophages following IFN-γ treatment was shown to be largely dependent on NO production (9, 10). Additionally, pharmacologic inhibition of NOS or genetic deletion of *Nos2* resulted in increased viral titers and mortality following EV infection in mice (9, 18). Moreover, NO affects several events in the late stages of the life cycle of VV, including viral DNA replication, viral protein synthesis, and virion maturation *in vitro* (32). These studies provided some of the first evidence that macrophage-produced NO has direct antiviral effects.

In addition to inhibiting HSV-1 replication *in vitro*, macrophage-derived NO has been shown to have anti-HSV properties *in vivo*. In a mouse model of HSV-1-mediated corneal disease, iNOS was highly induced in the trigeminal ganglion (TG) of HSV-1-infected mice, and its expression was markedly reduced in mice depleted of macrophages (22). Depletion of macrophages prior to HSV-1 infection resulted in markedly reduced iNOS expression and higher viral loads in the TG of infected mice (22, 23), suggesting that macrophages were the main source of iNOS expression in the affected tissues following HSV-1 infection and that NO had important anti-HSV-1 properties *in vivo*. Consistent with these data, inhibition of NOS activity resulted in increased viral loads in the TG (22). Additional studies showed that F4/80^+^GR-1^+^ inflammatory monocytes were recruited to the eye via an IFN-α-driven CCL2 gradient and restricted HSV-1 replication in that tissue via NO production (24). It was further shown that NO production by F4/80^+^ macrophages in the brains of HSV-1-infected mice blocked viral replication in a partially TLR2- and TLR9-dependent mechanism (25). Finally, following footpad inoculation, HSV-1-infected *Nos2*^−/−^ mice displayed a delayed clearance of virus from the dorsal root ganglia (DRG) and exhibited an increase in the frequency of virus reactivation in DRG (26).

The reactivity of NO and its higher oxides and nitrosothiol products (84) makes it likely that a variety of molecular targets are involved in its antiviral action. It has been shown that NO can inhibit ribonucleotide reductase (85, 86), a rate-limiting enzyme in DNA synthesis, and NO can lead to the deamination of mammalian and bacterial DNA (87, 88), which may be important antiviral mechanisms. Indeed, HSV-1 encodes its own ribonucleotide reductase and although it is not required for HSV-1 replication *in vitro*, it is necessary under conditions where the intracellular pool of deoxynucleotides is limited (89, 90). Thus, by inactivating this cellular and/or viral enzyme, NO may halt virus replication by directly inhibiting viral DNA synthesis.

In addition to HSV-1, treatment of primary human cells with an NO donor following infection with human cytomegalovirus (HCMV), a beta-herpesvirus, resulted in a significant reduction of early and late viral protein expression (28). Consistent with these *in vitro* data, *Nos2*^−/−^ mice (129/Sv/Ev x C57BL/6 F1) exhibited increased viral titers and mortality following infection with murine CMV (MCMV; Smith VR194 strain) (29).

Nitric oxide has also been shown to have antiviral properties on a chicken herpesvirus, Marek’s disease virus (MDV), which can cause T cell lymphomas in chickens: Addition of NO-generating compounds inhibited viral replication in chicken fibroblasts (33). Additionally, the treatment of chickens with an inhibitor of iNOS increased the level of MDV replication *in vivo* (34). Further studies demonstrated that NO production was limited to chickens that were genetically resistant to tumor development following MDV infection or to chickens that were vaccinated before being inoculated with MDV (35). Thus, NO appeared to be produced in both types of resistance to tumor development in Marek’s disease, either acquired after vaccination or genetic. Together, these findings suggest a role of NO in the protective immune mechanisms against Marek’s disease, possibly through its activity on viral replication.

Finally, studies with HBV, a hepadnavirus associated with acute and chronic hepatitis, demonstrated that HBV replicated to higher levels in the livers of HBV-transgenic *Nos2*^−/−^ mice than control transgenic mice, and transgenic *Nos2*^−/−^ mice had increased liver disease (36). It was further demonstrated that NO production by mononuclear cells, most likely macrophages, in the liver mediated most of the antiviral activity resulting from IFN-γ production by virus-specific T cells (36), suggesting an antiviral role for macrophage-derived NO following HBV infection in mice.

In addition to DNA viruses, macrophage-derived NO also exerts antiviral effects against a number of RNA viruses. Inhibition of JEV, a mosquito-transmitted flavivirus that causes encephalitis in humans, in IFN-γ-activated RAW 264.7 macrophages *in vitro* correlated with NO production, and IFN-γ-activated RAW 264.7 macrophage-mediated inhibition of JEV replication in murine neuroblastoma N18 cells was NO-dependent (12). Moreover, inhibition of NOS activity led to increased mortality in JEV-infected mice (12).

In terms of its mechanism of action, NO was found to inhibit JEV RNA synthesis, viral protein accumulation, and virus release from infected cells *in vitro* (12). These data suggest that NO may be directly or indirectly inhibiting viral enzymes and/or other cellular components required for viral replication, and this may subsequently block viral protein synthesis. Additionally, NO may interfere with the release and/or maturation of virions.

Monocyte/macrophage-derived NO may also block replication of DENV, another mosquito-transmitted flavivirus. Infection with DENV resulted in increased levels of NO in patients with dengue fever, the classic form of the disease (37). Additionally, iNOS expression was induced in CD14^+^ monocytes from a subset of acutely infected individuals (13). It was further shown that *ex vivo* infection of human monocytes with DENV-1 resulted in increased iNOS expression, and inhibition of iNOS activity led to increased DENV antigen detection in these cells (13). Moreover, treatment of C6/36 mosquito cells with an NO donor resulted in reduced DENV-positive cells (13). These data suggest that DENV replication is susceptible to NO-mediated inhibition. Consistent with this, *Nos2*^−/−^ mice were shown to be more susceptible to DENV infection, resulting in more severe disease and increased lethality in mouse models of DENV-2 and DENV-3 infection (38, 39). It was further demonstrated that, following DENV infection *in vivo*, IL-12 and IL-18 induced IFN-γ production, resulting in iNOS expression and NO production, which contributed to viral control (38, 39).

In addition to monocyte/macrophage-derived NO, a recent study demonstrated that platelets isolated from patients with dengue fever had increased l-arginine transport and increased NO production compared to platelets from healthy controls (40). However, NO has anti-aggregatory properties, and Mendes-Ribeiro et al. (40) found that dengue patients exhibited decreased collagen-induced platelet aggregation, consistent with the vascular leak and hemorrhagic manifestations of dengue hemorrhagic fever/dengue shock syndrome (DHF/DSS), thus establishing an association between reduced platelet aggregation, enhancement of the l-arginine–NO pathway, and DHF/DSS (41).

In contrast, Getts and colleagues showed that experimentally abrogating NO activity during West Nile virus (WNV) encephalitis, a related flavivirus, in NO-competent mice at a specific, relatively late time point prolonged survival of infected mice, while pharmacological inactivation throughout disease did not (42). Combined, these data suggest that although during DENV infection IFN-γ-induced NO production has a role in antiviral defense, it is likely that dysregulation of the IL-12/18–IFN-γ–NO axis leads to immune-mediated damage in certain flavivirus infections. Along these lines, it has also been shown that treatment of mice with a NOS inhibitor increased mortality rates following Sindbis virus (SINV) infection (43), suggesting a protective role for NO during this particular CNS infection. However, SINV replication in the brain was unaffected. Furthermore, treatment of neuroblastoma cells with NO donors had little effect on SINV replication but increased cell viability (43). These data suggest that NO protects mice from fatal SINV-induced encephalitis by a distinct mechanism that does not directly involve the inhibition of virus growth but rather may enhance survival of the infected neuron until the immune response can control virus replication.

Nitric oxide also plays an antiviral role during CNS infection with reovirus. Infection of neonatal mice with the prototypic neurotropic reovirus strain (T3A) induced iNOS expression in brain areas demonstrating reovirus antigen expression and associated virus-induced injury (44). Reovirus also induced iNOS expression following *in vitro* infection of primary neuronal and glial cultures. Reovirus was shown to infect a subpopulation of microglial cells *in vitro* (44), suggesting that direct virus interaction may induce iNOS in this specialized population of macrophages. Treatment of neuronal cultures with an NO donor inhibited viral replication whereas a NOS inhibitor increased viral growth (44), suggesting iNOS has the potential to exert antiviral activity *in vivo*.

Finally, coxsackievirus infection has been shown to induce expression of iNOS in macrophages infiltrating the hearts of infected mice (17). Treatment of WT mice with a NOS inhibitor and infection of *Nos2*^−/−^ mice resulted in more severe coxsackievirus-induced pancreatitis and myocarditis, elevated viral loads in tissues, and decreased survival compared to WT mice following coxsackievirus B3 (CVB3) infection (14, 15, 17). Similarly, *Nos2*^−/−^ mice infected with coxsackievirus B4 exhibited decreased survival and delayed viral clearance compared to WT mice (16). These data suggest an antiviral effect of NO against coxsackievirus infection. Consistent with this, it was demonstrated that NO inhibits the 2A and 3C proteinases of CVB3 *in vitro* (91). Additionally, CVB3-infected outbred mice showed significantly reduced signs of myocarditis after treatment with NO donors (91).

## NO Production Can be Detrimental to Host

### Myeloid cell production of NO can be immunopathologic during virus infections

Despite its protective capacity during some viral infections, NO can also contribute to immunopathology. The pathological effects of NO are likely due, at least in part, to oxidative damage caused by the interaction of NO with oxygen radicals such as the superoxide anion radical $\left(O_{2}^{-}\right)$ and hydrogen peroxide (H_2_O_2_).

For example, although addition of an NO donor to virus-infected MDCK cells reduced influenza A and B viral burden *in vitro* (45), treatment of mice with inhaled NO (iNO) did not decrease the viral load of influenza A (mouse-adapted H1N1 strain)-infected mice; in fact, prophylactic treatment with iNO resulted in enhanced weight loss and decreased survival following infection (46), suggesting a pathogenic role for NO. Consistent with this, chickens, which show a high level of mortality and associated pathology following avian influenza infection, had higher levels of iNOS expression in the lungs compared with H5N1 influenza-infected ducks, which show relatively minor symptoms following influenza infection (47). Additionally, Akaike and colleagues (48) found evidence of the production of peroxynitrite, which is generated through the reaction of NO and O_2_^–^, in the lungs of influenza A (mouse-adapted H2N2 strain)-infected mice. Moreover, inhibition of NOS resulted in enhanced survival and decreased pneumonia, but not decreased viral loads, in influenza-infected mice (48, 49), suggesting that NO was contributing to pathogenesis rather than having direct antiviral effects. *Nos2*^−/−^ mice also survived a lethal dose of influenza A virus (PR/8/34 strain) infection with little histopathologic evidence of pneumonitis; however, in these studies no infectious virus was detected in *Nos2*^−/−^ mice at day 6 after infection (49). The enhanced viral control in *Nos2*^−/−^ mice was shown to require the activity of IFN-γ (51), with *Nos2*^−/−^ mice also producing increased virus-specific IgG2a antibody titers (50). Additionally, genetic deletion of *Nos2* or pharmacologic inhibition of NOS enhanced survival of mice inoculated with the highly pathogenic (non-mouse-adapted) 1918 influenza virus strain, although mice exhibited similar viral loads to control mice in lung tissue at the peak of viral replication (51). Influenza infection *in vitro* was shown to induce apoptosis, and a reduction in influenza-mediated apoptosis was noted in cells treated with a NOS inhibitor (52). Similarly, fewer apoptotic cells were found in the lungs of influenza-infected *Nos2*^−/−^ mice, suggesting that NO mediates cell death following influenza infection (52). The cellular source of iNOS/NO following influenza infection in mice was shown to be CCR2^+^ inflammatory monocytes that accumulate in the lungs: CCR2^−/−^ mice survived a lethal challenge of influenza infection (PR/8/34 strain) and had significantly reduced accumulation of iNOS-expressing macrophages in the lung, with no associated increase in viral titers or dissemination (53).

It was also recently shown that a subset of monocyte-derived dendritic cells (DCs), described as TNF-α/iNOS-producing DCs (tipDCs), accumulate in greater numbers during the course of lethal versus sublethal influenza infections, suggesting a pathogenic role for this subpopulation of myeloid cells (54). Interestingly, though, Aldridge et al. (54) found that the tipDCs also stimulated a local, protective CD8^+^ T cell response in the virus-infected respiratory tract, indicating both protective and pathogenic roles for these cells in influenza infection. It was further shown that partially compromising tipDC recruitment via treatment with pioglitazone, a synthetic agonist of the peroxisome proliferator-activated receptor-γ (PPAR-γ), was protective against lethal influenza challenge (54). Pioglitazone treatment led to a reduction in the levels of CCL2 (MCP-1) and MCP-3 in the BAL fluid of influenza-infected mice (54). Pioglitazone has also been shown to reduce the production of a wide range of proinflammatory molecules, including iNOS (55), providing further evidence for the importance of NO production by monocyte-derived cells in the pathogenesis of influenza infection.

Pharmacologic inhibition of NOS using l-NMMA also decreased pneumonitis and increased survival following intranasal infection of CBA/J mice with HSV-1, despite a 17-fold increase in viral titers in the lung at day 3 after inoculation (58). In contrast, treatment of BALB/c mice with a different NOS inhibitor [aminoguanidine (AG), administered intranasally] resulted in enhanced pneumonitis, viral titers, and mortality following infection with a different strain of HSV-1 (59). Thus, the precise role of NO in HSV-1 pneumonitis remains to be determined. NO and other ROS/RNS were also shown to be pathogenic in the brains of mice with herpes encephalitis: iNOS was induced in CD11b^+^ resident microglia following intranasal infection with HSV-1, and oxidative and nitrative damage was found in the brains of infected animals (60).

A common neurological complication of HIV infection in the developed world is sensory neuronal injury accompanied by inflammation, which is clinically manifested as disabling pain and gait instability. Feline immunodeficiency virus (FIV) infection of cats, which causes similar neuroinflammation together with immunosuppression in cats, resulted in induction of iNOS and STAT-1, which were predominantly produced by macrophages, in DRG (64). Additionally, inhibition of NOS resulted in reduced nitrotyrosine and prevented neuronal injury in FIV-infected DRG cultures *in vitro* (64). These data suggest that lentivirus infection contributes to axonal and neuronal injury through a mechanism involving M1 macrophage immune activation mediated by STAT-1 and iNOS activation. In addition to these studies, infection of mice with the retrovirus LP-BM5, which causes profound immunodeficiency, induces CD11b^+^GR-1^+^Ly-6C^+^ MDSC-like cells that inhibit both T- and B-cell responses in an iNOS/NO-dependent but arginase-independent fashion (65). This study identified an important – and only recently appreciated – role for iNOS-expressing myeloid cell-mediated suppression of B cell responses in retrovirus infection.

### Myeloid cell production of NO can inhibit viral clearance

The oxidative effects of NO have also been shown to inhibit immune cells, particularly T cells. This phenomenon has been appreciated for a number of years in the context of tumors (92), where myeloid suppressor cells can inhibit the anti-tumor T cell response via the effects of NO in addition to other mechanisms (2, 4). In a similar manner, it has been shown that NO can inhibit the antiviral immune response.

MCMV clearance from BALB/c mice is predominantly CD8^+^ T cell-mediated. A recent report showed that MCMV infection in BALB/c mice induced CD11b^+^Ly-6C^hi^ inflammatory monocyte recruitment from the bone marrow to infected tissues that was dependent on CCR2 signaling (66). This recruitment was shown to inhibit antigen-specific CD8^+^ T cell activation, expansion, and cytotoxic activity via NO production, thus facilitating viral persistence (66).

In a similar fashion, NO may contribute to a defective immune response following infection of mice with an attenuated neurotropic coronavirus (rJ2.2 strain of mouse hepatitis virus). rJ2.2-infected WT mice exhibited mild acute encephalitis, followed by a non-lethal, chronic demyelinating disease (68). In marked contrast, rJ2.2 infection of mice that transgenically express CCL2 in the brain (CCL2 Tg) ineffectively cleared virus and rapidly succumbed to the infection (68). CCL2 Tg mice mounted a dysregulated immune response, characterized by increased accumulation of iNOS-expressing macrophages and microglia as well as regulatory T cells, but decreased Arg1 expression (68). These data suggest that persistent CCL2 overexpression establishes and sustains an immunological milieu that may predispose mice to a defective immune response to a typically minimally virulent virus.

## Arginase Activity Can be Beneficial for Tissue Repair Following Virus Infection

Arginase activity is important for wound healing and tissue regeneration through the production of polyamines and proline (2). In the context of some viral infections, arginase activity and M2 macrophage activation have been shown to be beneficial for tissue repair following virus-induced damage. For instance, resolution of severe respiratory syncytial virus (RSV)-induced bronchiolitis in mice is mediated by M2 macrophages that counteract cyclooxygenase (COX)-2-induced lung pathology (19, 20). Arg1 was induced in the lungs of RSV-infected mice, and its induction was shown to be IL-4Rα-dependent (19). Additionally, WT macrophages adoptively transferred into RSV-infected IL-4Rα^−/−^ mice restored the M2 phenotype in the lungs and decreased lung pathology (19). It was further shown that the lipoxogenase pathway was important for M2 macrophage activation and lung resolution following RSV infection (20). Most recently it was demonstrated that treating mice with agents that sustain Arg1 expression (e.g., IL-4/anti-IL-4 immune complexes) limited RSV-induced lung pathology (21).

Consistent with a pathogenic role for iNOS/NO following influenza infection (described above), it was recently shown that the presence of airway bacteria polarize alveolar macrophages into a M2 phenotype, thus limiting influenza-mediated lethal lung inflammation. Wang and colleagues (27) demonstrated that priming with *Staphylococcus aureus*, which commonly colonizes the upper respiratory mucosa, attenuated influenza-mediated lung injury via TLR2 signaling that recruited peripheral CCR2^+^CD11b^+^ monocytes into the alveoli (27). These monocytes polarized alveolar macrophages into a M2 phenotype characterized by high Arg1 as well as Ym1, FIZZ1, and IL-10 expression (27). It was further shown that *S. aureus*-primed M2 alveolar macrophages inhibited inflammatory cell recruitment to the lung, including neutrophils, NK cells, and CD8 T cells (27). *S. aureus*-primed M2 alveolar macrophages also expressed higher levels of the inhibitory ligand PD-L1 (27), suggesting that expression of a combination of anti-inflammatory cytokines and inhibitory ligands could be the mechanisms by which *S. aureus*-primed M2 alveolar macrophages limit influenza-mediated lung inflammation.

As discussed above, coxsackievirus B3 (CVB3) infection causes myocarditis in human beings as well as in male BALB/c mice. Although female mice do not develop severe myocarditis, both male and female mice have comparable numbers of infiltrating macrophages and viral titers in the heart following CVB3 infection (30). The macrophages infiltrating the heart in male mice were skewed toward a M1 phenotype characterized by high expression of iNOS (17) as well as M1-associated cytokines such as IFN-γ and IL-12 (30). Additionally, inhibition of NOS resulted in increased viral titers and higher mortality in CVB3-infected mice (17), consistent with an antiviral role for NO during CVB3 infection (see above). However, in contrast to male mice, the heart-infiltrating macrophages in female mice were skewed toward a M2 phenotype characterized by high expression of Arg1 as well as IL-4 and IL-10 (30). Moreover, adoptive transfer of *ex vivo*-programed M1 macrophages significantly increased myocarditis in both male and female mice. Strikingly, transfer of M2-programed macrophages into susceptible male mice alleviated myocardial inflammation by modulating the local cytokine profile from a M1 to M2 phenotype and promoting peripheral regulatory T cell (Treg) differentiation (30). Using different variants of CVB3, one that caused myocarditis in C57BL/6 mice and one that did not, it was additionally shown that the myocarditic variant induced a M1 macrophage phenotype (31). In contrast, the amyocarditic variant induced a M2 macrophage phenotype, which was also associated with the activation of NKT cells that promoted a Treg response (31). The ability of NKT cells to suppress myocarditis was shown by adoptive transfer of purified NKT cells into NKT knockout (Jα18 knockout) mice infected with the myocarditic CVB3 variant, which inhibited cardiac inflammation and increased Treg response (31). Cardiac virus titers were equivalent in all mouse strains indicating that NKT cells did not participate in control of virus infection (31). Thus, although NO appears to have antiviral properties against CVB3, these data indicate an important role for Arg1-expressing M2 macrophages in controlling CVB3-induced myocarditis.

## Arginase Activity Can Promote Viral Persistence and/or Exacerbated Immunopathology

### Arginase activity can inhibit viral clearance

As a consequence of their co-evolution with their hosts, viruses have developed numerous strategies to evade the host immune system and ensure their own replication and survival. Recent studies have identified a new evasion strategy for viruses: exploitation of the host’s anti-inflammatory, wound repair response to promote chronic infection.

Two strains of LCMV – Armstrong (Arm) and clone 13 (C13) – have been studied for decades as models for acute and chronic infections (93). Infection of mice with the Arm strain leads to a robust CD8^+^ T cell response that rapidly clears the virus (94), whereas infection with C13 results in T cells with impaired functionality, enabling the virus to persist (95). It was recently demonstrated that C13 infection led to an enhanced and sustained expansion of cells that resembled MDSCs (70). These suppressive myeloid cells inhibited T cell proliferation *ex vivo* via an iNOS/NO-dependent but Arg1-independent mechanism. Another study, however, found that Arg1-expressing immunoregulatory antigen presenting cells induced during C13 infection suppressed T cell responses (67). Most recently, it was demonstrated that T cell responses were improved – resulting in clearance of the normally chronic C13 infection – when either myeloid cells or T cells lacked IL-10 production (96). Overall, these data demonstrate the importance of iNOS/Arg1-expressing myeloid cells in viral persistence.

Similar to LCMV C13 infection, it was recently demonstrated that infection of mice with the arthritogenic alphaviruses Ross River virus (RRV) and chikungunya virus (CHIKV) resulted in the induction of Arg1 in macrophages in the infected and inflamed musculoskeletal tissues (69). It was further shown that genetic deletion of myeloid cell *Arg1* resulted in enhanced viral control in inflamed muscle tissue and reduced tissue pathology following RRV infection in mice (69), suggesting an important role for Arg1-expressing macrophages in the persistence of these chronic viruses.

Infection of mice with Theiler’s murine encephalomyelitis virus (TMEV) results in persistent virus infection in the CNS, which contributes to the development of a demyelinating disease that has similarities with multiple sclerosis. Bowen and Olson (97) showed that CD11b^+^Ly-6C^+^ cells infiltrated the CNS following infection and were the dominant cell type during the innate immune response. Depletion of the CD11b^+^Ly-6C^+^ cells via administration of an anti-GR-1 Ab resulted in reduced development of demyelinating disease and enhanced virus-specific CD4^+^ and CD8^+^ T cell responses (97). Additionally, TMEV-infected, anti-GR-1 Ab-treated mice had decreased myelin-specific CD4^+^ T cell responses compared to control Ab-treated mice during the demyelinating disease at a later time post-infection (97). Although the expression of Arg1 was not investigated in this study, TMEV-infected mice had elevated expression of IL-10 in the brain and spinal cord (97), suggesting a role for this cytokine in the suppression of antiviral T cell responses, potentially through the effects of Arg1.

Interestingly, a role for the modulation of arginine metabolism in viral control versus persistence along with associated disease has recently been demonstrated for the tumor-inducing, chicken-specific herpesvirus MDV. We mentioned above that MDV was vulnerable to the antiviral properties of NO, with iNOS being induced in genetically resistant chickens and in vaccinated chickens (35). In contrast, MDV induced strong macrophage arginase activity in cell extracts from adherent monocytes from genetically susceptible chickens, but not in chickens that were resistant to Marek’s disease, either genetically or acquired after vaccination (35). Together, these data suggest that in the case of Marek’s disease, the state of resistance versus sensitivity to disease was correlated with a reciprocal balance of NOS versus arginase activities in macrophages.

This phenomenon of Arg1-mediated T cell suppression has also been recognized in human viral infections. Arg1 mRNA and protein levels were elevated in HCV-infected liver cell lines *in vitro* and in HCV-infected liver samples compared with paired hepatocellular carcinoma samples from the same patients or with uninfected liver tissues (71). Additionally, the number of MDSCs in chronic HCV patients correlated with levels of plasma HCV-RNA (98). Cai et al. (98) also found that MDSCs from patients with chronic HCV infection suppressed T cell function via an Arg1-dependent mechanism. An additional study found that more PBMCs from chronic HCV patients expressed the phenotypic markers of MDSCs than PBMCs from healthy controls, and these cells expressed increased levels of p47^phox^, a component of the NADPH oxidase complex (99), suggesting a role for ROS in MDSC-mediated suppression. Consistent with this, CD33^+^ mononuclear cells co-cultured with HCV-infected hepatocytes or HCV core protein suppressed T cell proliferation in a ROS-dependent manner (99). Overall, these data suggest that multiple mechanisms – including arginine metabolism and ROS – may be at play in myeloid cell-mediated suppression of anti-HCV T cell responses.

It has been suggested that prolonged immune activation during chronic virus infections, such as HCV and HIV, provides an environment that drives viral replication and disease progression (100, 101). Moreover, immune activation can drive an anti-inflammatory response to limit immunopathology, which can be characterized by the presence of M2-like macrophages. Indeed, similar to HCV infection, a role for arginase and M2-polarized MDSC-like cells has been identified in the suppression of antiviral T cell responses following HIV infection. Individuals with detectable HIV-1 infection showed an increase in the frequency of CD163^+^CD16^+^CD14^+^ monocytes, which are thought to be precursors of M2 macrophages, when compared to seronegative or HIV-1-infected persons with undetectable viral loads, and monocyte frequency correlated positively with HIV-1 viremia and negatively with CD4^+^ T cell counts (in patients with counts <450 cells/μl) (72). Furthermore, Qin and colleagues (73) observed elevated levels of MDSCs, defined as HLA-DR^−/low^ CD11b^+^CD33^+/high^CD14^+^CD15^−^cells, in the peripheral blood of HIV-1-seropositive subjects compared with healthy controls, and these MDSCs suppressed T cell responses in an Arg1-dependent manner. Moreover, PBMCs from HIV-seropositive patients exhibited increased levels of arginase activity (73). Cloke and colleagues (74) found that increased arginase activity correlated with lower CD4^+^ T cell counts, and this association was abrogated following antiretroviral treatment (75). Additionally, exposure of PBMCs to HIV gp120 expanded T cell-suppressive MDSCs *in vitro* (76). These data point to a direct role for arginase-expressing MDSC-like cells in the suppression of anti-HIV T cell responses. Consistent with that, individuals co-infected with HIV and *Leishmania* parasites had increased arginase activity in PBMCs and plasma compared with *Leishmania*-only infected individuals, even though *Leishmania* infection alone results in increased arginase activity (77). In addition, the parasite load in the spleen was significantly higher in co-infected patients (77). The arginase-expressing cells were identified as low-density granulocytes (77). These results suggest that increased arginase might contribute to the poor immune responses and disease outcome characteristic of patients with *Leishmania* and HIV co-infection.

Hepatitis B virus (HBV) infection is another common chronic viral infection, with estimates as high as 350 million chronically infected humans (102). Bility and colleagues (78) recently developed a humanized mouse model with both a human immune system and human liver cells, named the A2/NSG-hu HSC/Hep humanized mouse model, to study the pathogenesis of HBV infection. Following HBV infection, the mice developed persistent HBV infection as well as chronic hepatitis and liver fibrosis (78). The liver disease was associated with a high level of infiltrating human macrophages with a M2-like activation phenotype (78). Similarly, M2-like macrophage accumulation was seen in chronic HBV-infected patients, and M2-like macrophage induction in the liver was associated with accelerated liver fibrosis and necrosis in patients with acute HBV-induced liver failure (78), suggesting a role for M2 macrophages in persistent HBV infection. Additionally, patients with acute HBV infection had increased serum levels of arginase, and this serum inhibited IFN-γ production by CD8^+^ T cells (79). Das et al. (80) also found decreased l-arginine levels in the circulation of chronic HBV patients with marked liver inflammation (>100 ALT) and increased arginase activity in liver extracts taken directly *ex vivo* from patients with chronic HBV compared with those from patients with other types of liver pathology (80). They further showed that CD8^+^ T cells from chronic HBV patients, regardless of their antigen specificity, exhibited less IL-2 but not IFN-γ or TNF-α production and impaired proliferation following TCR-dependent stimulation, indicating an aberrant antiviral T cell response in chronic HBV infection (80). In the A2/NSG-hu HSC/Hep humanized mouse model, HBV-infected mice had impaired liver T cell responses, and M2 macrophages were associated with T cells in the liver (78). Expression of the TCR signaling molecule CD3ζ was reduced in both peripheral and intrahepatic CD8^+^ T cells from chronic HBV patients; similarly, CD28 was also downregulated on CD8^+^ T cells from high viral load HBV patients (80). Downregulation of the CD3ζ molecule has previously been shown to occur in the arginine-depleted tumor microenvironment. Consistent with this, *in vitro* transfection of CD3ζ and CD28 restored IL-2 production and supplementation of l-arginine partially restored CD3ζ expression and T cell proliferation (80). These data suggest a role for arginase activity and arginine depletion in the impairment of anti-HBV T cells functions.

In the absence of iNKT cells, influenza A (PR/8 strain) infection was shown to induce the expansion of CD11b^+^GR-1^+^ MDSCs in the lungs of mice, which suppressed influenza-specific T cell and antibody responses through the activity of both arginase and NOS, resulting in higher viral titers and increased mortality (81). Adoptive transfer of iNKT cells reversed this phenotype; mice had an increased survival rate, reduced viral titers, and increased virus-specific immune responses, suggesting a novel immunomodulatory role for iNKT cells during influenza virus infection (81). Moreover, these authors identified that influenza infection in humans induced the expansion of CD11b^+^ myeloid cells with suppressive activity that could be reduced by iNKT cell activation or the inhibition of arginase and NOS activity. Similarly, it was recently shown that highly pathogenic H5N1 and H1N1 influenza virus infection induced the accumulation of CD11b^+^GR-1^+^ cells and the expression of Arg1 in the lungs (82), further supporting a role for M2-polarized MDSC-like cells in promoting viral persistence and immunopathology.

Helminth infection induces the expression of type 2 cytokines and is associated with M2 macrophage activation, as determined by Arg1, FIZZ1, and Ym1 expression. Indeed, Osborne and colleagues (83) found that Arg1, FIZZ1, and Ym1 were highly induced in the ileum of mice infected with the helminth *Trichinella spiralis* (Ts). Interestingly, they further showed that co-infection of mice with Ts and murine norovirus (MNV) resulted in decreased frequencies and numbers of MNV-specific CD8^+^ and CD4^+^ T cells within the small intestine and spleen as well as decreased polyfunctionality of these T cells, compared to Ts-only infected mice (83). Additionally, the defective T cell responses were associated with increased viral loads in the double-infected mice compared to the mono-infected controls (83), suggesting that Ts-elicited M2-activated macrophages inhibited the antiviral T cell response to MNV. Lastly, neutralization of Ym1, a chitinase-like molecule, in co-infected mice partially restored antiviral immunity and was associated with enhanced control of viral replication (83). These data point to a new mechanism by which Arg1-expressing macrophages inhibit antiviral responses.

Cumulatively, these data are reminiscent of macrophages found in tumors (e.g., MDSCs, TAMs) that have been shown to suppress anti-tumor T cell responses via a variety of NO- and/or Arg1-dependent mechanisms (4, 5). Indeed, in a mouse model of human papillomavirus (HPV)-induced cancer, Arg1-expressing CD11b^+^F4/80^+^ macrophages infiltrated the tumors and inhibited T cell responses, including virus-specific T cells, by suppressing T cell proliferation and promoting a regulatory phenotype (103). Moreover, depletion of the tumor-infiltrating macrophages resulted in reduced tumor growth and increased tumor infiltration by virus-specific CD8^+^ T cells (103). Thus, increasing evidence points to a direct role for arginase-expressing M2-polarized cells in the suppression of antiviral T cell responses and the persistence of a variety of important pathogenic viruses. In addition to the actions of iNOS and Arg1, MDSC-like cells can employ other mechanisms to promote chronic viral infections, which were recently reviewed by Goh and colleagues (104).

### M2 macrophage activation can promote immunopathology

In contrast to some parasitic infections where M2 macrophages limit Th2 cell-mediated immunopathology, M2-polarized macrophages have been shown to promote immunopathology in some viral infections. For example, it was recently demonstrated that SARS-CoV infection of mice induced suppressive alveolar macrophages that inhibited the induction of antiviral T cell responses, a phenotype that was reversed by the adoptive transfer of activated bone marrow-derived DCs into mice prior to virus infection (56). Additionally, SARS-CoV-infected mice lacking hematopoietic STAT-1 expression were shown to have greater weight loss and lung pathology, and this was associated with the activation of M2 macrophages (57). To further test the role of M2 macrophages in enhanced pathogenesis following SARS-CoV infection, the authors generated STAT-1/STAT-6 double knockout mice due to the established role for STAT-6 in driving M2 macrophage activation in response to IL-4/IL-13 stimulation. STAT-1/STAT-6 double knockout mice, which reversed the upregulation of M2 macrophages observed in STAT-1-deficient mice, had reduced lung disease and prefibrotic lesions (57). These data support the notion that M2 macrophages contribute to SARS-CoV pathogenesis.

In another example, mice deficient in the IFN-γR exhibit more severe disease following infection with murine gamma-herpesvirus-68 (MHV-68), including interstitial and intra-alveolar fibrosis that is reminiscent of idiopathic pulmonary fibrosis (IPF) in human beings. In this model, alveolar macrophages were recruited to the lungs of MHV-68-infected IFN-γR^−/−^ mice, were associated with areas of fibrosis, and exhibited a M2-polarized phenotype characterized by the expression of FIZZ1, Ym1, and Arg1 (61). Additionally, lung tissue from patients with IPF showed increased expression of Arg1 in alveolar macrophages compared with normal lung (61). These results suggest that virus-induced upregulation of Arg1 could be mediating lung fibrogenesis. MHV-68 infection in IFN-γR^−/−^ mice also resulted in fibrosis in lymphoid tissues such as the spleen, which is a site of latent MHV-68 infection, and the liver (62, 63). Similar to the lung, MHV-68 infection in the absence of IFN-γR signaling induced a M2 macrophage response in the spleen, characterized by high Arg1 expression along with FIZZ1 and M2/Th2 cytokines such as IL-13, resulting in fibrotic disease in the spleen (105). Moreover, depletion of T cells prevented MHV-68-mediated fibrosis in IFN-γR^−/−^ mice (62), suggesting that M2 macrophages were further driving Th2 activation to possibly create a M2/Th2 cytokine-induced cycle, resulting in the exaggerated pathology. In contrast to IFN-γR^−/−^ mice, iNOS was induced in the spleen of MHV-68-infected WT mice (105), indicating an important role for IFN-γ in inducing a M1-associated immune response to control gamma-herpesvirus infection and limiting Arg1-mediated immunopathology.

## Conclusion

Macrophages and other myeloid cells have marked phenotypic heterogeneity, as a result of distinct cellular differentiation programs, distribution in tissues, and responsiveness to various endogenous and exogenous stimuli. Indeed, macrophages have well-established roles in development, tissue homeostasis, coordinating the adaptive immune response and inflammation, as well as directing tissue resolution and repair following damage – processes that are often modulated via the actions of the arginine-hydrolyzing enzymes Nos2 and Arg1. We have highlighted a number of viral infections in which these enzymes have a beneficial effect: NO has antiviral properties against a variety of viruses, and arginase activity can mediate tissue repair and regeneration following a viral insult (Table 1). However, NO production can also result in immunopathology in some virus infections, and the suppressive functions of Arg1-expressing macrophages can promote immunopathology. Additionally, some viruses have exploited the immune-suppressive properties of iNOS- and/or Arg1-expressing macrophages to evade the immune response, particularly the antiviral T cell response, resulting in chronic viral infections.

Clearly, iNOS- and/or Arg1-mediated responses are important in many viral infections. Thus, there is the potential to develop the means to selectively stimulate or inhibit either M1 or M2 responses to mediate viral clearance or repair tissue damage. Due to the overlap in immunosuppressive mechanisms of iNOS- and/or Arg1-expressing suppressor cells, therapeutic strategies under development to limit the immunosuppressive effects of myeloid cells in cancer may be beneficial in treating persistent/chronic virus infections. However, as described above, iNOS and Arg1 activity can be both beneficial and detrimental during certain viral infections. Therefore, further research is needed to define the molecular and tissue-specific mechanism(s) by which iNOS and Arg1 influence the clearance of viral pathogens as well as the injury and repair of tissues. In addition, a better understanding of the pathways regulating macrophage polarization (specifically iNOS and/or Arg1 induction and activity), macrophage trafficking, and the precise effects of iNOS and Arg1 activity on other immune cells following different virus infections will inform the development of therapeutics that target critical effector molecules to promote viral control and limit immunopathology.

## Conflict of Interest Statement

The authors declare that the research was conducted in the absence of any commercial or financial relationships that could be construed as a potential conflict of interest.
